# Azido­(1,1-diphenyl­methanimine-κ*N*)[hydridotris(pyrazolyl-κ*N* ^2^)borato](triphenyl­phosphine-*κP*)ruthenium(II) diethyl ether solvate

**DOI:** 10.1107/S1600536808036039

**Published:** 2008-11-08

**Authors:** Chia-Her Lin, Ting-Shen Kuo, Hung-Chun Tong, Chih-Yung Chen Hsu, Yih-Hsing Lo

**Affiliations:** a Department of Chemistry, Chung-Yuan Christian University, Chung-Li 320, Taiwan; b Department of Chemistry, National Normal Taiwan University, Taipei 106, Taiwan; c Department of Chemical Engineering, Tatung University, Taipei 104, Taiwan

## Abstract

The reaction of [RuCl(C_9_H_10_BN_6_)(C_18_H_15_P)_2_] with benzo­phenone imine in methanol, in the presence of sodium azide, leads to the formation of the title compound, [Ru(C_9_H_10_BN_6_)(N_3_)(HN=CPh_2_)(C_18_H_15_P)]·C_4_H_10_O, which crystallizes as the diethyl ether solvate. In the crystal structure, the Ru atom is coordinated by three N atoms of one hydridotris(pyrazoly)borate anion, one P atom of one triphenyl­phosphine ligand, one N atom of the azide anion and one N atom of the benzophenone­imine ligand in a slightly distorted octa­hedral geometry. The azide anion is almost linear [177.0 (5)°], with an Ru—N—N angle of 125.9 (3)°. There is a small difference between the N—N distances [1.200 (5) and 1.164 (5) Å], the longer bond being adjacent to the Ru atom.

## Related literature

For general background, see: Agrell (1971 ▶); Alcock *et al.* (1992 ▶); Burrows *et al.* (2001 ▶); Moloy & Petersen (1995 ▶); Pavlik *et al.* (2005 ▶); Slugovc *et al.* (1997 ▶); Trofimenko *et al.* (1993 ▶). For related structures, see: Dori & Ziolo (1973 ▶); Gemel *et al.* (1996 ▶); Meyer *et al.* (1998 ▶); Huynh *et al.* (2003 ▶); Slugovc *et al.* (1998 ▶).
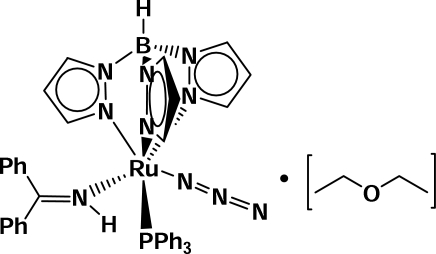


## Experimental

### 

#### Crystal data

[Ru(C_9_H_10_BN_6_)(N_3_)(C_13_H_11_N)(C_18_H_15_P)]·C_4_H_10_O
*M* *_r_* = 873.76
Triclinic, 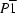

*a* = 11.7387 (12) Å
*b* = 13.0535 (13) Å
*c* = 14.7187 (15) Å
α = 70.445 (2)°
β = 81.716 (2)°
γ = 88.040 (3)°
*V* = 2102.9 (4) Å^3^
*Z* = 2
Mo *K*α radiation
μ = 0.46 mm^−1^
*T* = 200 (2) K
0.19 × 0.07 × 0.02 mm

#### Data collection

Nonius KappaCCD diffractometer
Absorption correction: multi-scan (Blessing, 1995 ▶) *T*_min_ = 0.918, *T*_max_ = 0.989
16858 measured reflections
7382 independent reflections
4895 reflections with *I* > 2σ(*I*)
*R*_int_ = 0.061

#### Refinement

*R*[*F* ^2^ > 2σ(*F* ^2^)] = 0.050
*wR*(*F* ^2^) = 0.113
*S* = 1.01
7382 reflections
523 parameters
H-atom parameters constrained
Δρ_max_ = 1.75 e Å^−3^
Δρ_min_ = −0.56 e Å^−3^

### 

Data collection: *COLLECT* (Nonius, 1999 ▶); cell refinement: *HKL* *DENZO* and *SCALEPACK* (Otwinowski & Minor 1997 ▶); data reduction: *HKL* *DENZO* and *SCALEPACK*; program(s) used to solve structure: *SHELXS97* (Sheldrick, 2008 ▶); program(s) used to refine structure: *SHELXL97* (Sheldrick, 2008 ▶); molecular graphics: *ORTEP-3 for Windows* (Farrugia, 1997 ▶); software used to prepare material for publication: *WinGX* publication routines (Farrugia, 1999 ▶).

## Supplementary Material

Crystal structure: contains datablocks I, global. DOI: 10.1107/S1600536808036039/nc2120sup1.cif

Structure factors: contains datablocks I. DOI: 10.1107/S1600536808036039/nc2120Isup2.hkl

Additional supplementary materials:  crystallographic information; 3D view; checkCIF report

## Figures and Tables

**Fig. 1.**
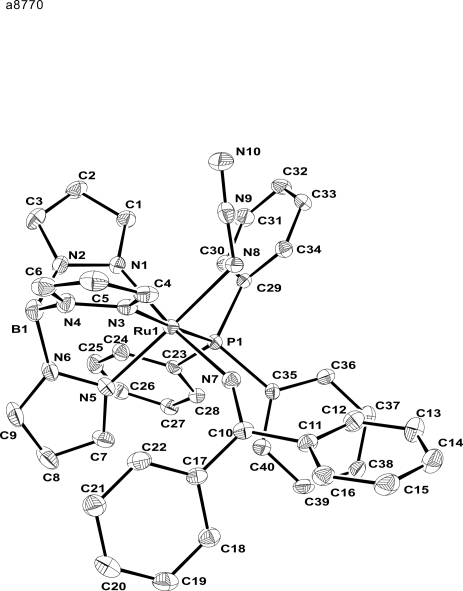
Molecular structure of (the title compound with labelling and displacement ellipsoids drawn at the 30% probability level (H atoms are shown as spheres of arbitrary radius).

## supplementary crystallographic information

### Comment

The hydridotris(pyrazoly)borate anion (Tp,HB(pz)_3_) has been used by Trofimenko as a ligand in various transition metal complexes (Trofimenko,1993). Ruthenium(II) hydridotripyrazolylborate complexes, Ru(Tp), are of interest for stoichiometric and catalytic transformations of organic molecules (Pavlik *et al.*, 2005). The complex [Ru(Tp)Cl(PPh_3_)_2_] (Alock *et al.*, 1992) has been used as the starting material for the synthesis of several complexes because the chloride atom and the PPh_3_ group can be easily substituted (Slugovc *et al.*, 1997; Moloy & Petersen, 1995; Burrows, 2001). On the other hand, the azide anion N_3_^-^ is a versatile ligand because it shows a variety of coordination modes and compounds with this ligand shows interesting thermal and photochemical reactivities (Dori & Ziolo, 1973; Meyer *et al.*, 1998; Huynh *et al.*, 2003).

In the crystal structure of the title compound, the environment about the ruthenium metal center corresponds to a slightly distorted octahedron and the bite angle of the Tp ligand leads to an average N—Ru1—N angle of 86.3°, which is only slightly distorted from 90° (Fig. 1). The three Ru1—N(Tp) bond lengths of 2.077 (3), 2.114 (4), and 2.084 (4) Å) are slightly longer than the average distance of 2.038 Å observed in other ruthenium Tp complexes (Gemel *et al.* 1996; Slugovc *et al.* 1998). The Ru1—N7 and N7—C10 bond lengths of 2.053 (3) and 1.304 (5) Å correspond to a single Ru—N and a double C=N bond. The angles around C10 of 122.3 (4)°, 118.6 (4)° and 119.1 (4)° indicate a *sp*^2^ hybridization.

The azide anion is almost linear (177.0 (5)°) and is coordinated to Ru with an Ru—N(8)—N(9) angle of 125.9 (3)°. There is a small difference between the N—N distances [1.200 (5) and 1.164 (5) Å], the longer being adjacent to the Ru atom. It is also noted the title complex shows a ν_as_(N_3_) stretching band in a lower energy region, at 2036 cm^-1^,compared with the typical values of these bands in azido complexes (2120–2030 cm^-1^; Agrell, 1971).

### Experimental

To a solution of [Ru(Tp)Cl(PPh_3_)_2_] (3.95 g, 4.50 mmol) in CH_3_OH (100 ml), an excess of benzophenoneimine (7.9 ml, 45.0 mmol) and NaN_3_ (2.93 g, 45.0 mmol) were added and the solution was refluxed for 120 min. Afterwards the reaction mixture was concentrated to approximately 10 ml and cooled to 253 K. The yellow precipitate which has formed was filtered off, washed with CH_2_Cl_2_and was dried under reduced pressure to give the title compound (2.34 g, 65% yield). The bright-yellow crystals used for X-ray structure analysis were obtained within 3 days by slow diffusion of diethyl ether into a solution of the title compound in CH_2_Cl_2_ at 273 K.

### Refinement

The H atoms were placed in idealized positions and constrained to ride on their parent atoms, with C—H = 0.95 - 0.99 Å and *U*_iso_(H) = 1.2 or 1.5*U*_eq_(C), B—H = 1.0 Å and *U*_iso_(H) = 1.2*U*_eq_(B), and N—H = 0.88 Å and *U*_iso_(H) = 1.2*U*_eq_(N).

### Crystal data

**Table:**

[Ru(C_9_H_10_BN_6_)(N_3_)(C_13_H_11_N)(C_18_H_15_P)]·C_4_H_10_O	*Z* = 2
*M**_r_* = 873.76	*F*_000_ = 904
Triclinic, *P*1	*D*_x_ = 1.380 Mg m^−^^3^
Hall symbol: -P 1	Mo *K*α radiation λ = 0.71073 Å
*a* = 11.7387 (12) Å	Cell parameters from 16922 reflections
*b* = 13.0535 (13) Å	θ = 2.4–22.8º
*c* = 14.7187 (15) Å	µ = 0.46 mm^−^^1^
α = 70.445 (2)º	*T* = 200 (2) K
β = 81.716 (2)º	Prism, red
γ = 88.040 (3)º	0.19 × 0.07 × 0.02 mm
*V* = 2102.9 (4) Å^3^	

### Data collection

**Table:**

Nonius KappaCCD diffractometer	7382 independent reflections
Radiation source: fine-focus sealed tube	4895 reflections with *I* > 2σ(*I*)
Monochromator: graphite	*R*_int_ = 0.061
*T* = 200(2) K	θ_max_ = 25.0º
CCD rotation images, thick slices scans	θ_min_ = 1.5º
Absorption correction: multi-scan(Blessing, 1995)	*h* = −10→13
*T*_min_ = 0.918, *T*_max_ = 0.989	*k* = −13→15
16858 measured reflections	*l* = −16→17

### Refinement

**Table:**

Refinement on *F*^2^	Secondary atom site location: difference Fourier map
Least-squares matrix: full	Hydrogen site location: inferred from neighbouring sites
*R*[*F*^2^ > 2σ(*F*^2^)] = 0.050	H-atom parameters constrained
*wR*(*F*^2^) = 0.113	*w* = 1/[σ^2^(*F*_o_^2^) + (0.0454*P*)^2^] where *P* = (*F*_o_^2^ + 2*F*_c_^2^)/3
*S* = 1.01	(Δ/σ)_max_ = 0.001
7382 reflections	Δρ_max_ = 1.75 e Å^−^^3^
523 parameters	Δρ_min_ = −0.56 e Å^−^^3^
Primary atom site location: structure-invariant direct methods	Extinction correction: none

### Special details

**Table:**

Geometry. All e.s.d.'s (except the e.s.d. in the dihedral angle between two l.s. planes) are estimated using the full covariance matrix. The cell e.s.d.'s are taken into account individually in the estimation of e.s.d.'s in distances, angles and torsion angles; correlations between e.s.d.'s in cell parameters are only used when they are defined by crystal symmetry. An approximate (isotropic) treatment of cell e.s.d.'s is used for estimating e.s.d.'s involving l.s. planes.
Refinement. Refinement of *F*^2^ against ALL reflections. The weighted *R*-factor *wR* and goodness of fit *S* are based on *F*^2^, conventional *R*-factors *R* are based on *F*, with *F* set to zero for negative *F*^2^. The threshold expression of *F*^2^ > σ(*F*^2^) is used only for calculating *R*-factors(gt) *etc*. and is not relevant to the choice of reflections for refinement. *R*-factors based on *F*^2^ are statistically about twice as large as those based on *F*, and *R*- factors based on ALL data will be even larger.

### Fractional atomic coordinates and isotropic or equivalent isotropic displacement parameters (Å^2^)

**Table:**

	*x*	*y*	*z*	*U*_iso_*/*U*_eq_	
B1	0.8157 (5)	0.8932 (4)	0.7251 (4)	0.0386 (15)	
H1'	0.8125	0.9641	0.7376	0.046*	
C1	0.8247 (4)	0.8636 (4)	0.4879 (3)	0.0354 (12)	
H1	0.8285	0.8213	0.4462	0.043*	
C2	0.8292 (4)	0.9767 (4)	0.4569 (4)	0.0424 (13)	
H2	0.8359	1.0254	0.3918	0.051*	
C3	0.8219 (4)	1.0027 (4)	0.5398 (4)	0.0427 (13)	
H3	0.8236	1.0743	0.5426	0.051*	
C4	1.0523 (4)	0.7072 (4)	0.7588 (3)	0.0367 (12)	
H4	1.0886	0.6421	0.7554	0.044*	
C5	1.1011 (4)	0.7847 (4)	0.7874 (3)	0.0426 (13)	
H5	1.1747	0.7834	0.8073	0.051*	
C6	1.0193 (4)	0.8638 (4)	0.7806 (3)	0.0405 (13)	
H6	1.0270	0.9292	0.7942	0.049*	
C7	0.6070 (4)	0.6735 (4)	0.8423 (3)	0.0395 (13)	
H7	0.5763	0.6032	0.8535	0.047*	
C8	0.5601 (5)	0.7450 (4)	0.8884 (4)	0.0517 (15)	
H8	0.4938	0.7336	0.9363	0.062*	
C9	0.6287 (5)	0.8348 (4)	0.8508 (4)	0.0464 (14)	
H9	0.6189	0.8990	0.8677	0.056*	
C10	0.8495 (4)	0.4452 (3)	0.8627 (3)	0.0290 (11)	
C11	0.8971 (4)	0.3351 (3)	0.8753 (3)	0.0315 (11)	
C12	0.8898 (4)	0.2849 (4)	0.8067 (4)	0.0390 (12)	
H12	0.8505	0.3203	0.7527	0.047*	
C13	0.9382 (5)	0.1845 (4)	0.8150 (4)	0.0497 (14)	
H13	0.9317	0.1516	0.7673	0.060*	
C14	0.9959 (5)	0.1326 (4)	0.8926 (4)	0.0495 (15)	
H14	1.0297	0.0639	0.8986	0.059*	
C15	1.0041 (4)	0.1810 (4)	0.9615 (4)	0.0470 (14)	
H15	1.0442	0.1453	1.0148	0.056*	
C16	0.9548 (4)	0.2809 (4)	0.9540 (3)	0.0374 (12)	
H16	0.9603	0.3126	1.0027	0.045*	
C17	0.8004 (4)	0.4727 (3)	0.9494 (3)	0.0298 (11)	
C18	0.7310 (4)	0.3970 (4)	1.0255 (3)	0.0362 (12)	
H18	0.7154	0.3280	1.0211	0.043*	
C19	0.6847 (4)	0.4219 (4)	1.1073 (4)	0.0461 (14)	
H19	0.6373	0.3698	1.1585	0.055*	
C20	0.7062 (4)	0.5207 (4)	1.1154 (4)	0.0475 (14)	
H20	0.6737	0.5374	1.1716	0.057*	
C21	0.7757 (5)	0.5958 (4)	1.0410 (4)	0.0484 (14)	
H21	0.7913	0.6645	1.0461	0.058*	
C22	0.8228 (4)	0.5716 (4)	0.9591 (3)	0.0417 (13)	
H22	0.8712	0.6236	0.9087	0.050*	
C23	0.5401 (4)	0.6507 (3)	0.6241 (3)	0.0265 (10)	
C24	0.5229 (4)	0.7597 (4)	0.6139 (3)	0.0334 (11)	
H24	0.5853	0.8036	0.6149	0.040*	
C25	0.4153 (4)	0.8042 (4)	0.6023 (3)	0.0389 (12)	
H25	0.4046	0.8792	0.5933	0.047*	
C26	0.3236 (4)	0.7410 (4)	0.6035 (3)	0.0371 (12)	
H26	0.2497	0.7722	0.5958	0.045*	
C27	0.3386 (4)	0.6325 (4)	0.6157 (3)	0.0352 (12)	
H27	0.2752	0.5884	0.6172	0.042*	
C28	0.4466 (4)	0.5878 (3)	0.6259 (3)	0.0313 (11)	
H28	0.4568	0.5129	0.6343	0.038*	
C29	0.7223 (4)	0.6166 (3)	0.4899 (3)	0.0281 (11)	
C30	0.6531 (4)	0.6761 (3)	0.4225 (3)	0.0352 (12)	
H30	0.5815	0.7024	0.4443	0.042*	
C31	0.6870 (5)	0.6977 (4)	0.3238 (3)	0.0422 (13)	
H31	0.6388	0.7389	0.2783	0.051*	
C32	0.7903 (5)	0.6598 (4)	0.2913 (4)	0.0428 (13)	
H32	0.8144	0.6762	0.2235	0.051*	
C33	0.8588 (4)	0.5977 (4)	0.3579 (4)	0.0377 (12)	
H33	0.9287	0.5689	0.3359	0.045*	
C34	0.8257 (4)	0.5776 (3)	0.4555 (3)	0.0299 (11)	
H34	0.8742	0.5364	0.5006	0.036*	
C35	0.6572 (4)	0.4502 (3)	0.6762 (3)	0.0253 (10)	
C36	0.6827 (4)	0.3772 (3)	0.6266 (3)	0.0337 (12)	
H36	0.7174	0.4026	0.5605	0.040*	
C37	0.6578 (4)	0.2664 (4)	0.6728 (4)	0.0381 (12)	
H37	0.6774	0.2170	0.6383	0.046*	
C38	0.6057 (4)	0.2287 (4)	0.7670 (4)	0.0401 (13)	
H38	0.5869	0.1536	0.7976	0.048*	
C39	0.5806 (4)	0.3003 (4)	0.8176 (3)	0.0400 (13)	
H39	0.5448	0.2743	0.8834	0.048*	
C40	0.6073 (4)	0.4101 (4)	0.7731 (3)	0.0360 (12)	
H40	0.5912	0.4584	0.8092	0.043*	
C41	0.3203 (7)	0.0075 (7)	0.9844 (5)	0.120 (3)	
H41A	0.2559	−0.0211	1.0365	0.180*	
H41B	0.3228	0.0870	0.9648	0.180*	
H41C	0.3926	−0.0218	1.0078	0.180*	
C42	0.3049 (6)	−0.0244 (6)	0.9012 (5)	0.092 (2)	
H42A	0.3008	−0.1047	0.9212	0.110*	
H42B	0.2315	0.0047	0.8779	0.110*	
C43	0.3815 (7)	−0.0031 (5)	0.7392 (5)	0.088 (2)	
H43A	0.3098	0.0316	0.7164	0.105*	
H43B	0.3744	−0.0822	0.7518	0.105*	
C44	0.4811 (6)	0.0426 (5)	0.6632 (5)	0.086 (2)	
H44A	0.4699	0.0298	0.6031	0.128*	
H44B	0.5517	0.0073	0.6856	0.128*	
H44C	0.4874	0.1210	0.6505	0.128*	
N1	0.8145 (3)	0.8235 (3)	0.5848 (3)	0.0284 (9)	
N2	0.8118 (3)	0.9105 (3)	0.6168 (3)	0.0306 (9)	
N3	0.9472 (3)	0.7371 (3)	0.7369 (2)	0.0291 (9)	
N4	0.9270 (3)	0.8335 (3)	0.7515 (3)	0.0327 (9)	
N5	0.7002 (3)	0.7161 (3)	0.7804 (3)	0.0299 (9)	
N6	0.7130 (3)	0.8180 (3)	0.7856 (3)	0.0338 (9)	
N7	0.8548 (3)	0.5152 (3)	0.7752 (3)	0.0303 (9)	
H7A	0.8889	0.4811	0.7365	0.036*	
N8	0.9577 (3)	0.6233 (3)	0.5978 (3)	0.0336 (10)	
N9	1.0225 (3)	0.6845 (3)	0.5335 (3)	0.0347 (10)	
N10	1.0865 (4)	0.7402 (3)	0.4696 (4)	0.0632 (15)	
O1	0.3970 (3)	0.0154 (3)	0.8253 (3)	0.0591 (10)	
P1	0.68709 (10)	0.59726 (9)	0.62088 (8)	0.0255 (3)	
Ru1	0.82112 (3)	0.66797 (3)	0.68486 (3)	0.02500 (12)	

### Atomic displacement parameters (Å^2^)

**Table:**

	*U*^11^	*U*^22^	*U*^33^	*U*^12^	*U*^13^	*U*^23^
B1	0.045 (4)	0.035 (3)	0.041 (4)	0.000 (3)	−0.006 (3)	−0.019 (3)
C1	0.045 (3)	0.033 (3)	0.025 (3)	−0.002 (2)	−0.004 (2)	−0.006 (2)
C2	0.056 (4)	0.030 (3)	0.030 (3)	−0.003 (2)	−0.005 (3)	0.004 (2)
C3	0.050 (3)	0.023 (3)	0.050 (4)	0.000 (2)	−0.010 (3)	−0.003 (2)
C4	0.032 (3)	0.041 (3)	0.026 (3)	0.000 (2)	0.000 (2)	0.000 (2)
C5	0.032 (3)	0.061 (3)	0.031 (3)	−0.011 (3)	−0.006 (2)	−0.010 (3)
C6	0.041 (3)	0.050 (3)	0.030 (3)	−0.018 (3)	−0.002 (2)	−0.013 (3)
C7	0.033 (3)	0.054 (3)	0.033 (3)	−0.011 (3)	0.004 (2)	−0.018 (3)
C8	0.040 (3)	0.072 (4)	0.052 (4)	−0.004 (3)	0.008 (3)	−0.040 (3)
C9	0.046 (3)	0.054 (3)	0.054 (4)	0.004 (3)	−0.003 (3)	−0.040 (3)
C10	0.023 (3)	0.032 (3)	0.028 (3)	−0.002 (2)	−0.005 (2)	−0.004 (2)
C11	0.026 (3)	0.030 (3)	0.034 (3)	−0.003 (2)	−0.002 (2)	−0.005 (2)
C12	0.040 (3)	0.034 (3)	0.039 (3)	0.001 (2)	−0.010 (3)	−0.007 (2)
C13	0.062 (4)	0.031 (3)	0.057 (4)	−0.001 (3)	−0.005 (3)	−0.017 (3)
C14	0.053 (4)	0.032 (3)	0.055 (4)	0.008 (3)	−0.004 (3)	−0.007 (3)
C15	0.048 (3)	0.043 (3)	0.038 (3)	0.010 (3)	−0.007 (3)	0.003 (3)
C16	0.041 (3)	0.038 (3)	0.028 (3)	0.005 (2)	−0.006 (2)	−0.004 (2)
C17	0.029 (3)	0.033 (3)	0.024 (3)	−0.002 (2)	−0.006 (2)	−0.003 (2)
C18	0.031 (3)	0.036 (3)	0.037 (3)	−0.002 (2)	−0.003 (2)	−0.007 (2)
C19	0.039 (3)	0.052 (3)	0.038 (3)	−0.006 (3)	0.007 (3)	−0.007 (3)
C20	0.045 (3)	0.065 (4)	0.034 (3)	0.002 (3)	0.000 (3)	−0.020 (3)
C21	0.062 (4)	0.047 (3)	0.038 (3)	−0.013 (3)	0.000 (3)	−0.018 (3)
C22	0.049 (3)	0.041 (3)	0.031 (3)	−0.013 (3)	−0.002 (3)	−0.005 (2)
C23	0.030 (3)	0.031 (2)	0.017 (2)	0.002 (2)	−0.001 (2)	−0.008 (2)
C24	0.034 (3)	0.037 (3)	0.034 (3)	−0.002 (2)	−0.004 (2)	−0.016 (2)
C25	0.044 (3)	0.034 (3)	0.039 (3)	0.013 (2)	−0.009 (3)	−0.012 (2)
C26	0.029 (3)	0.048 (3)	0.033 (3)	0.007 (2)	−0.003 (2)	−0.014 (3)
C27	0.030 (3)	0.045 (3)	0.032 (3)	−0.004 (2)	0.000 (2)	−0.017 (2)
C28	0.036 (3)	0.028 (2)	0.026 (3)	0.000 (2)	0.000 (2)	−0.007 (2)
C29	0.037 (3)	0.020 (2)	0.025 (3)	−0.004 (2)	0.001 (2)	−0.007 (2)
C30	0.038 (3)	0.034 (3)	0.032 (3)	0.000 (2)	−0.005 (2)	−0.009 (2)
C31	0.053 (4)	0.043 (3)	0.027 (3)	0.001 (3)	−0.009 (3)	−0.006 (2)
C32	0.057 (4)	0.045 (3)	0.023 (3)	−0.010 (3)	0.008 (3)	−0.011 (2)
C33	0.038 (3)	0.038 (3)	0.036 (3)	−0.002 (2)	0.009 (3)	−0.016 (2)
C34	0.033 (3)	0.032 (3)	0.025 (3)	−0.002 (2)	−0.005 (2)	−0.010 (2)
C35	0.025 (2)	0.024 (2)	0.028 (3)	0.0003 (19)	−0.004 (2)	−0.011 (2)
C36	0.039 (3)	0.028 (3)	0.028 (3)	−0.004 (2)	0.000 (2)	−0.004 (2)
C37	0.046 (3)	0.030 (3)	0.042 (3)	−0.003 (2)	−0.005 (3)	−0.019 (2)
C38	0.051 (3)	0.024 (3)	0.043 (3)	−0.005 (2)	−0.008 (3)	−0.007 (2)
C39	0.052 (3)	0.038 (3)	0.022 (3)	−0.009 (3)	0.001 (2)	−0.003 (2)
C40	0.046 (3)	0.032 (3)	0.028 (3)	−0.004 (2)	0.002 (2)	−0.010 (2)
C41	0.107 (7)	0.195 (9)	0.076 (6)	−0.029 (6)	0.016 (5)	−0.079 (6)
C42	0.066 (5)	0.120 (6)	0.096 (6)	−0.022 (4)	0.010 (4)	−0.052 (5)
C43	0.117 (6)	0.081 (5)	0.077 (5)	−0.035 (4)	−0.011 (5)	−0.040 (4)
C44	0.135 (7)	0.058 (4)	0.061 (5)	−0.026 (4)	−0.006 (5)	−0.017 (3)
N1	0.030 (2)	0.024 (2)	0.030 (2)	0.0015 (17)	−0.0039 (18)	−0.0082 (18)
N2	0.038 (2)	0.022 (2)	0.032 (2)	−0.0003 (17)	−0.0050 (19)	−0.0090 (18)
N3	0.028 (2)	0.032 (2)	0.023 (2)	−0.0001 (18)	−0.0013 (18)	−0.0033 (18)
N4	0.040 (2)	0.029 (2)	0.030 (2)	−0.0026 (19)	−0.0062 (19)	−0.0100 (18)
N5	0.028 (2)	0.036 (2)	0.029 (2)	0.0026 (18)	−0.0040 (18)	−0.0154 (19)
N6	0.034 (2)	0.036 (2)	0.037 (2)	0.0011 (19)	−0.003 (2)	−0.021 (2)
N7	0.031 (2)	0.030 (2)	0.029 (2)	−0.0027 (17)	−0.0010 (18)	−0.0105 (19)
N8	0.035 (2)	0.028 (2)	0.031 (2)	0.0002 (19)	0.008 (2)	−0.0052 (19)
N9	0.030 (2)	0.036 (2)	0.044 (3)	0.006 (2)	−0.007 (2)	−0.021 (2)
N10	0.051 (3)	0.050 (3)	0.074 (4)	−0.013 (2)	0.029 (3)	−0.014 (3)
O1	0.061 (3)	0.061 (2)	0.062 (3)	0.004 (2)	−0.009 (2)	−0.030 (2)
P1	0.0300 (7)	0.0231 (6)	0.0225 (7)	0.0018 (5)	−0.0013 (5)	−0.0075 (5)
Ru1	0.0269 (2)	0.0245 (2)	0.0208 (2)	0.00069 (15)	−0.00016 (16)	−0.00537 (16)

### Geometric parameters (Å, °)

**Table:**

B1—N4	1.529 (7)	C25—H25	0.9500
B1—N2	1.541 (6)	C26—C27	1.374 (6)
B1—N6	1.548 (6)	C26—H26	0.9500
B1—H1'	1.0000	C27—C28	1.384 (6)
C1—N1	1.333 (5)	C27—H27	0.9500
C1—C2	1.392 (6)	C28—H28	0.9500
C1—H1	0.9500	C29—C30	1.386 (6)
C2—C3	1.362 (6)	C29—C34	1.393 (6)
C2—H2	0.9500	C29—P1	1.845 (4)
C3—N2	1.344 (5)	C30—C31	1.384 (6)
C3—H3	0.9500	C30—H30	0.9500
C4—N3	1.333 (5)	C31—C32	1.377 (7)
C4—C5	1.386 (6)	C31—H31	0.9500
C4—H4	0.9500	C32—C33	1.384 (7)
C5—C6	1.374 (6)	C32—H32	0.9500
C5—H5	0.9500	C33—C34	1.371 (6)
C6—N4	1.337 (5)	C33—H33	0.9500
C6—H6	0.9500	C34—H34	0.9500
C7—N5	1.323 (5)	C35—C36	1.384 (6)
C7—C8	1.385 (6)	C35—C40	1.390 (6)
C7—H7	0.9500	C35—P1	1.840 (4)
C8—C9	1.353 (7)	C36—C37	1.398 (6)
C8—H8	0.9500	C36—H36	0.9500
C9—N6	1.342 (5)	C37—C38	1.363 (6)
C9—H9	0.9500	C37—H37	0.9500
C10—N7	1.299 (5)	C38—C39	1.378 (6)
C10—C17	1.473 (6)	C38—H38	0.9500
C10—C11	1.487 (6)	C39—C40	1.387 (6)
C11—C12	1.389 (6)	C39—H39	0.9500
C11—C16	1.395 (6)	C40—H40	0.9500
C12—C13	1.384 (6)	C41—C42	1.454 (8)
C12—H12	0.9500	C41—H41A	0.9800
C13—C14	1.376 (7)	C41—H41B	0.9800
C13—H13	0.9500	C41—H41C	0.9800
C14—C15	1.378 (7)	C42—O1	1.411 (7)
C14—H14	0.9500	C42—H42A	0.9900
C15—C16	1.385 (6)	C42—H42B	0.9900
C15—H15	0.9500	C43—O1	1.405 (7)
C16—H16	0.9500	C43—C44	1.483 (8)
C17—C22	1.383 (6)	C43—H43A	0.9900
C17—C18	1.395 (6)	C43—H43B	0.9900
C18—C19	1.382 (6)	C44—H44A	0.9800
C18—H18	0.9500	C44—H44B	0.9800
C19—C20	1.369 (7)	C44—H44C	0.9800
C19—H19	0.9500	N1—N2	1.366 (4)
C20—C21	1.380 (7)	N1—Ru1	2.077 (3)
C20—H20	0.9500	N3—N4	1.354 (5)
C21—C22	1.382 (6)	N3—Ru1	2.114 (4)
C21—H21	0.9500	N5—N6	1.372 (5)
C22—H22	0.9500	N5—Ru1	2.084 (4)
C23—C28	1.384 (6)	N7—Ru1	2.056 (3)
C23—C24	1.389 (6)	N7—H7A	0.8800
C23—P1	1.840 (4)	N8—N9	1.200 (5)
C24—C25	1.382 (6)	N8—Ru1	2.097 (4)
C24—H24	0.9500	N9—N10	1.164 (5)
C25—C26	1.373 (6)	P1—Ru1	2.3070 (13)
			
N4—B1—N2	107.3 (4)	C30—C31—H31	119.9
N4—B1—N6	108.1 (4)	C31—C32—C33	119.7 (5)
N2—B1—N6	107.8 (4)	C31—C32—H32	120.1
N4—B1—H1'	111.1	C33—C32—H32	120.1
N2—B1—H1'	111.1	C34—C33—C32	120.0 (5)
N6—B1—H1'	111.1	C34—C33—H33	120.0
N1—C1—C2	110.0 (4)	C32—C33—H33	120.0
N1—C1—H1	125.0	C33—C34—C29	121.2 (4)
C2—C1—H1	125.0	C33—C34—H34	119.4
C3—C2—C1	105.3 (4)	C29—C34—H34	119.4
C3—C2—H2	127.3	C36—C35—C40	118.1 (4)
C1—C2—H2	127.3	C36—C35—P1	123.5 (3)
N2—C3—C2	108.8 (4)	C40—C35—P1	118.4 (3)
N2—C3—H3	125.6	C35—C36—C37	120.7 (4)
C2—C3—H3	125.6	C35—C36—H36	119.7
N3—C4—C5	110.2 (5)	C37—C36—H36	119.7
N3—C4—H4	124.9	C38—C37—C36	120.5 (4)
C5—C4—H4	124.9	C38—C37—H37	119.7
C6—C5—C4	104.7 (4)	C36—C37—H37	119.7
C6—C5—H5	127.6	C37—C38—C39	119.5 (4)
C4—C5—H5	127.6	C37—C38—H38	120.3
N4—C6—C5	108.7 (4)	C39—C38—H38	120.3
N4—C6—H6	125.6	C38—C39—C40	120.5 (4)
C5—C6—H6	125.6	C38—C39—H39	119.8
N5—C7—C8	111.0 (5)	C40—C39—H39	119.8
N5—C7—H7	124.5	C39—C40—C35	120.7 (4)
C8—C7—H7	124.5	C39—C40—H40	119.6
C9—C8—C7	105.3 (5)	C35—C40—H40	119.6
C9—C8—H8	127.4	C42—C41—H41A	109.5
C7—C8—H8	127.4	C42—C41—H41B	109.5
N6—C9—C8	108.7 (4)	H41A—C41—H41B	109.5
N6—C9—H9	125.7	C42—C41—H41C	109.5
C8—C9—H9	125.7	H41A—C41—H41C	109.5
N7—C10—C17	122.0 (4)	H41B—C41—H41C	109.5
N7—C10—C11	118.8 (4)	O1—C42—C41	110.5 (6)
C17—C10—C11	119.2 (4)	O1—C42—H42A	109.6
C12—C11—C16	117.9 (4)	C41—C42—H42A	109.6
C12—C11—C10	120.9 (4)	O1—C42—H42B	109.6
C16—C11—C10	121.2 (4)	C41—C42—H42B	109.6
C13—C12—C11	121.7 (5)	H42A—C42—H42B	108.1
C13—C12—H12	119.2	O1—C43—C44	109.7 (6)
C11—C12—H12	119.2	O1—C43—H43A	109.7
C14—C13—C12	119.7 (5)	C44—C43—H43A	109.7
C14—C13—H13	120.1	O1—C43—H43B	109.7
C12—C13—H13	120.1	C44—C43—H43B	109.7
C13—C14—C15	119.5 (5)	H43A—C43—H43B	108.2
C13—C14—H14	120.2	C43—C44—H44A	109.5
C15—C14—H14	120.2	C43—C44—H44B	109.5
C14—C15—C16	121.0 (5)	H44A—C44—H44B	109.5
C14—C15—H15	119.5	C43—C44—H44C	109.5
C16—C15—H15	119.5	H44A—C44—H44C	109.5
C15—C16—C11	120.2 (5)	H44B—C44—H44C	109.5
C15—C16—H16	119.9	C1—N1—N2	106.6 (3)
C11—C16—H16	119.9	C1—N1—Ru1	134.0 (3)
C22—C17—C18	118.2 (4)	N2—N1—Ru1	118.9 (3)
C22—C17—C10	121.9 (4)	C3—N2—N1	109.2 (4)
C18—C17—C10	119.9 (4)	C3—N2—B1	129.8 (4)
C19—C18—C17	120.4 (4)	N1—N2—B1	120.3 (4)
C19—C18—H18	119.8	C4—N3—N4	106.8 (4)
C17—C18—H18	119.8	C4—N3—Ru1	133.6 (3)
C20—C19—C18	120.9 (5)	N4—N3—Ru1	119.6 (3)
C20—C19—H19	119.6	C6—N4—N3	109.5 (4)
C18—C19—H19	119.6	C6—N4—B1	131.0 (4)
C19—C20—C21	119.3 (5)	N3—N4—B1	119.2 (4)
C19—C20—H20	120.3	C7—N5—N6	105.6 (4)
C21—C20—H20	120.3	C7—N5—Ru1	136.8 (3)
C20—C21—C22	120.3 (5)	N6—N5—Ru1	117.6 (3)
C20—C21—H21	119.9	C9—N6—N5	109.5 (4)
C22—C21—H21	119.9	C9—N6—B1	129.2 (4)
C21—C22—C17	121.0 (4)	N5—N6—B1	121.3 (4)
C21—C22—H22	119.5	C10—N7—Ru1	149.3 (3)
C17—C22—H22	119.5	C10—N7—H7A	105.4
C28—C23—C24	118.6 (4)	Ru1—N7—H7A	105.4
C28—C23—P1	121.3 (3)	N9—N8—Ru1	125.9 (3)
C24—C23—P1	119.7 (3)	N10—N9—N8	176.9 (5)
C25—C24—C23	120.1 (4)	C43—O1—C42	113.3 (5)
C25—C24—H24	120.0	C23—P1—C35	100.84 (19)
C23—C24—H24	120.0	C23—P1—C29	100.2 (2)
C26—C25—C24	120.6 (4)	C35—P1—C29	103.21 (19)
C26—C25—H25	119.7	C23—P1—Ru1	118.60 (15)
C24—C25—H25	119.7	C35—P1—Ru1	116.39 (14)
C25—C26—C27	119.9 (5)	C29—P1—Ru1	115.06 (14)
C25—C26—H26	120.0	N7—Ru1—N1	170.95 (14)
C27—C26—H26	120.0	N7—Ru1—N5	99.27 (14)
C26—C27—C28	119.7 (5)	N1—Ru1—N5	88.06 (14)
C26—C27—H27	120.1	N7—Ru1—N8	79.20 (14)
C28—C27—H27	120.1	N1—Ru1—N8	92.89 (14)
C23—C28—C27	121.0 (4)	N5—Ru1—N8	173.15 (15)
C23—C28—H28	119.5	N7—Ru1—N3	90.87 (14)
C27—C28—H28	119.5	N1—Ru1—N3	84.22 (14)
C30—C29—C34	118.1 (4)	N5—Ru1—N3	86.64 (14)
C30—C29—P1	122.1 (4)	N8—Ru1—N3	86.71 (15)
C34—C29—P1	119.6 (3)	N7—Ru1—P1	90.93 (10)
C31—C30—C29	120.8 (5)	N1—Ru1—P1	93.73 (10)
C31—C30—H30	119.6	N5—Ru1—P1	94.99 (10)
C29—C30—H30	119.6	N8—Ru1—P1	91.71 (11)
C32—C31—C30	120.1 (5)	N3—Ru1—P1	177.35 (10)
C32—C31—H31	119.9		
